# RAB9A Plays an Oncogenic Role in Human Liver Cancer Cells

**DOI:** 10.1155/2020/5691671

**Published:** 2020-04-30

**Authors:** Pengfei Sun, Lei Li, Zhongchao Li

**Affiliations:** Hepatobiliary and Pancreatic Surgery, Shandong Cancer Hospital and Institute, Shandong Fist Medical University and Shandong Academy of Medical Sciences, No. 440 Jiyan Road, Huaiyin District, Jinan City, 250117 Shandong Province, China

## Abstract

**Background:**

RAB9, as a member of the Rab GTPase family, is required for the transport of the mannose-6-phosphate receptor (MPR) from late endosomes to *trans*-Golgi network (TGN). However, the role of RAB9A in tumors, including liver cancer, is still unknown.

**Methods:**

We used pcDNA3.1 plasmid to upregulate the expression of RAB9A in Hep3b cells and used specific shRNA to downregulate the expression of RAB9A in HepG2 cells. Biological functions of RAB9A were performed by CCK-8 assay, colony formation assay, apoptosis analysis, transwell assays, and wound healing assays. Finally, an in-depth mechanism study was performed by western blot.

**Results:**

RAB9A promoted the proliferation and clonality of Hep3b and HepG2 cells. RAB9A also inhibited apoptosis and the activation of mitochondrial apoptotic pathway. In addition, RAB9A promoted the invasion and migration of Hep3b and HepG2 cells. Importantly, RAB9A activated the AKT/mTOR signaling pathway in human liver cancer cells. A double-effect inhibitor (BEZ235) significantly hindered the effect of RAB9A overexpression on the proliferation and invasion of Hep3b cells.

**Conclusion:**

Our data suggest that RAB9A plays a carcinogenic role in human liver cancer progression partially through AKT signaling pathways, suggesting that RAB9A may serve as a potential therapeutic target for liver cancer therapy.

## 1. Introduction

The occurrence and development of liver cancer is the most serious consequence of uncontrolled liver regeneration [1]. At present, primary liver cancer is an incurable disease [2]. Its annual mortality rate is about 100%, which means that most cases cannot survive for one year [3]. Conventional treatments include chemotherapy, arterial embolization, surgical resection, and radiofrequency ablation. However, the recurrence rate after receiving treatment is still as high as 70% or more. Therefore, it is necessary and urgent to explore the specific molecular mechanisms of the occurrence and development of liver cancer.

RAB9 is a member of the Rab GTPase family [4]. Members of this family are primarily located on different organelle membranes and together with their specific effectors to play a central role in regulating vesicle-mediated transport [5]. RAB9 is mainly located in the late endosomal membrane and is required for the transport of the mannose-6-phosphate receptor (MPR) from late endosomes to *trans*-Golgi network (TGN) [6]. In addition, RAB9 plays an important role in lysosomal biogenesis and late endosomal morphology [7]. It can also serve as a cellular target for certain pathogens and play an important role in pathogenic infections [8].

RAB9 has two isoforms, RAB9A and RAB9B. One study has shown that Rab9 is abnormally highly expressed in breast cancer tissues and plays critical roles in the biological action of breast cancer cells [9]. Rab9 is considered a good candidate for a new breast cancer treatment strategy. In addition, RAB9A knockdown can inhibit the proliferation, migration, and invasion of melanoma cells and induce apoptosis [10]. These data indicate that RAB9A may be involved in the regulation of tumor progression. However, the role of RAB9A in other tumors, including liver cancer, is still unknown.

In this study, we examined the effects of RAB9A on the biological function of hepatoma cells by function-gaining and function-losing experiments. Finally, we found that RAB9A plays a procancer role in liver cancer cells by affecting the activation of the AKT/mTOR signaling pathway.

## 2. Materials and Methods

### 2.1. Cell Culture

Human liver cancer cell lines, Hep3b and HepG2, were purchased from the Cell Bank of the Chinese Academy of Sciences (Shanghai, China). The cells were cultured in Dulbecco's modified Eagle's medium (DMEM, Hyclone, Logan, UT) supplemented with 10% FBS (Gibco, Grand Island, NY) at 37°C in 5% CO_2_.

### 2.2. Plasmid, shRNAs, and Transfection

The RAB9A cDNA sequence was cloned into the pcDNA3.1 vector. And recombinant plasmid was transfected into Hep3b cells to upregulate RAB9A expression (RAB9A-OV). shRNAs were synthesized and transfected into HepG2 cells to downregulate RAB9A expression (RAB9A-KD) (Ruibo, Guangzhou, China). Transfection of shRNA and plasmid was performed by Lipofectamine 2000 (Invitrogen, USA).

### 2.3. Western Blot

After transfection for 48 h, cell lysates were prepared by using RIPA buffer. The protein separation was performed by using 10% SDS-PAGE gel electrophoresis, which was followed by transmembrane onto a PVDF membrane. After blocking in 5% nonfat milk, the protein bands were washed with TBST buffer and incubated with primary antibodies at 4°C overnight, followed by incubation with the corresponding secondary antibodies for 1 h at room temperature. The visualization of protein bands was performed by using Pierce ECL Western Blotting Substrate (Thermo Scientific).

### 2.4. CCK-8 Assay

The cells were seeded into a 96-well plate with 3000 cells per well. 10 *μ*l CCK-8 solution was added into each well at different time points (24 h, 48 h, and 72 h). After incubation for 2 h, the absorbance at 450 nm was detected by a microplate reader.

### 2.5. Colony Formation Assay

Cells were seeded in a 6 cm petri dish at 500 cells and normally cultured for 14 days. Then the colonies were fixed with methanol and stained with 0.1% crystal violet (Sigma-Aldrich, St. Louis, MO). Visible colonies were photographed and counted manually.

### 2.6. Flow Cytometry Detection for Cell Apoptosis

Transfected cells were stained using an Annexin V-FITC Apoptosis Detection Kit I (BD Biosciences). Then, cells were analyzed through BD FACS Canto II (BD Biosciences) and analyzed with BD FACSDiva software. Cells were discriminated into viable cells, dead cells, early apoptosis cells, and apoptosis cells.

### 2.7. Transwell Assay

Cell invasion was evaluated by using Matrigel-coated Transwell chambers (BD Biosciences). 1 × 10^5^ cells in 200 *μ*l of DMEM medium containing no serum were added into the upper chamber. The lower chamber was added with 500 *μ*l of DMEM medium containing 10% FBS. Then, the cells were normally cultured for 24 h. Invaded cells on the lower surface of the chamber were stained with 0.1% crystal violet for 10 min. Cell number was counted under a microscope (Nikon TE2000).

### 2.8. Wound Healing Assays

Cells were cultured to the appropriate confluence in 6-well plates. The wound was created using sterile plastic tips. After washing with PBS, cells were cultured for 24 h with serum-free medium. Images were taken using a microscope. An average of five random widths of each wound was measured for quantitation.

### 2.9. Enzyme-Linked Immunosorbent Assay (ELISA)

ELISA was used to measure the concentrations of matrix metalloproteinase (MMP) 2 (cat. no. ab100606; Abcam, Cambridge, UK) and MMP9 (cat. no. ab100610; Abcam) in cell lysates. The samples from cells were collected after inoculation for 48 h. All experiments followed the manufacturer's protocol. The total protein concentration was normalized to the concentration in the sample before transfection.

### 2.10. Statistical Analysis

All the data analysis was performed by using Prism 7 (GraphPad Software). All experiments were performed in triplicate. Comparison between two groups was performed using unpaired two-tailed Student's *t*-test. *P* < 0.05 was considered statistically significant.

## 3. Results

### 3.1. RAB9A Promotes the Proliferation and Colony Formation of Human Liver Cancer Cells

**Figure 1:**
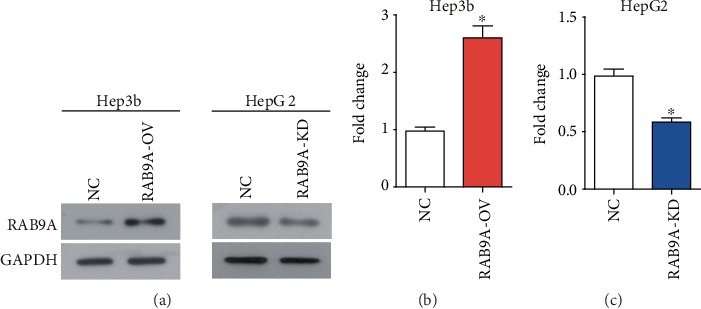
The protein expression of RAB9A was changed in Hep3b and HepG2 cells. After plasmid and shRNA were transfected into Hep3b (a and b) and HepG2 (a and c) cells, respectively, RAB9A protein expression was detected by western blot. ^∗^*P* < 0.05.

**Figure 2:**
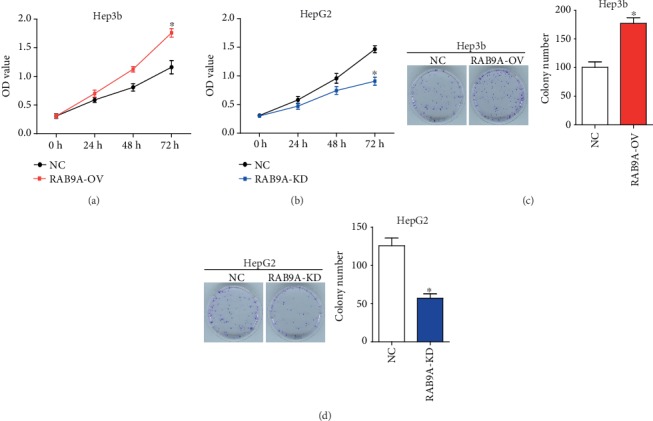
RAB9A promotes the proliferation and colony formation of human liver cancer cells. Plasmid and shRNA were transfected into Hep3b and HepG2 cells, respectively. Cell proliferation of Hep3b (a) and HepG2 (b) cells was detected by CCK8 assay. Clonality of Hep3b (c) and HepG2 (d) cells was detected by colony formation assay.

In order to investigate the biological function of RAB9A in human liver cancer, loss- and gain-of-function assays were performed. The protein expression of RAB9A was significantly upregulated in Hep3b cells (Figures 1(a) and 1(b), *P* < 0.05), and was downregulated in HepG2 cells (Figure 1(a) and 1(c), *P* < 0.05). The results of CCK8 assay indicated that overexpression of RAB9A significantly promoted the proliferation of Hep3b cells (Figure 2(a), *P* < 0.05) and RAB9A knockdown obviously inhibited the proliferation of HepG2 cells (Figure 2(b), *P* < 0.05). Furthermore, colony cell number was also significantly increased when RAB9A was overexpressed (Figure 2(c)) and was decreased when silence of RAB9A (Figure 2(d)).

### 3.2. RAB9A Inhibits Cell Apoptosis and Mitochondrial Apoptotic Pathway Activation in Human Liver Cancer Cells

**Figure 3:**
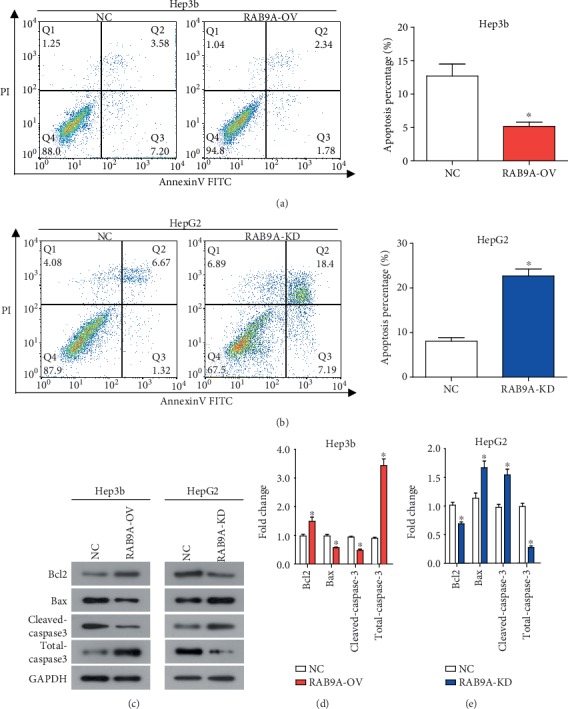
RAB9A inhibits cell apoptosis and mitochondrial apoptotic pathway activation in human liver cancer cells. The apoptosis percentage was detected through Annexin V/PI staining and flow cytometry in Hep3b (a) and HepG2 (b) cells. (c–e) Protein expression of Bcl2, Bax, cleaved caspase 3, and total caspase 3 was detected by western blot. ^∗^*P* < 0.05.

Cell apoptosis results are shown in Figures 3(a) and 3(b), which suggested that the apoptosis percentage of Hep3b cells was significantly decreased when RAB9A was overexpressed and the apoptosis percentage of HepG2 cells was significantly increased when RAB9A silencing. Overexpression of RAB9A also led to a significant downregulation of proapoptotic protein Bax and down-stream effector cleaved caspase 3, while upregulation of antiapoptotic protein Bcl2 and down-stream effector total caspase 3 (Figures 3(c) and 3(d)). When RAB9A was downregulated, the opposite result was obtained (Figures 3(c) and 3(e)). These data suggested that RAB9A inhibited the mitochondrial apoptotic pathway activation in Hep3b and HepG2 cells.

### 3.3. RAB9A Promotes Cell Invasion and Migration

**Figure 4:**
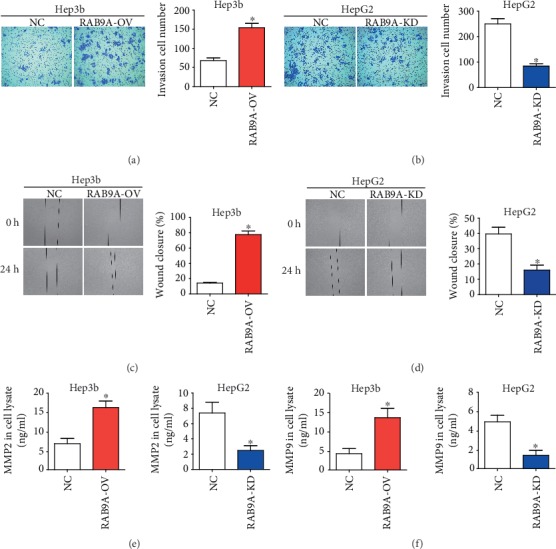
RAB9A promotes cell invasion and migration. The invasion of Hep3b (a) and HepG2 (b) cells was determined by transwell assay. The migration of Hep3b (c) and HepG2 (d) cells was determined by wound healing assay. The expression levels of MMP2 (e) and MMP9 (f) were measured by ELISA. ^∗^*P* < 0.05.

We continued to investigate whether RAB9A could affect the cell invasion and migration of human liver cancer cells. As showed in transwell assays, the invasion ability of Hep3b cells was obviously upregulated when RAB9A was overexpressed (Figure 4(a)), and the invasion ability of HepG2 cells was obviously downregulated when RAB9A silencing (Figure 4(b)). In addition, the results of wound healing assay showed that when RAB9A was silenced in HepG2 cells, the healing wound width was dwarfed compared to NC cells (Figure 4(d)). When RAB9A was overexpressed, the opposite result was obtained (Figure 4(c)). MMP2 and MMP9 are the two critical MMPs that mediate tumor cell invasion. Unsurprisingly, the overexpression of RAB9A induced the expression of MMP2 and MMP9 in Hep3b cells; while the deletion of RAB9A suppressed the expression of MMP2 and MMP9 in HepG2 cells (Figures 4(e) and 4(f)).

### 3.4. RAB9A Promotes the Malignant Behavior of Liver Cancer Cells by Activating the AKT/mTOR Signaling Pathway

**Figure 5:**
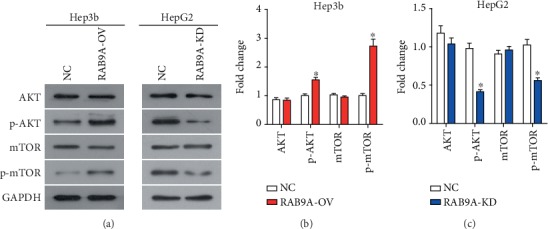
RAB9A promotes the activation of the AKT/mTOR signaling pathway. AKT signaling pathway components were detected (a) and quantified (b and c) by western blot. All experiments were performed in triplicate. ^∗^*P* < 0.05.

In order to investigate the action mechanism underlying the procancer effect of RAB9A in human liver cancer cells, we proposed that the AKT/mTOR signaling pathway might be associated with the biological function of RAB9A. As a result, overexpression of RAB9A led to increased phosphorylation level of AKT and mTOR proteins (Figure 5), and RAB9A knockdown led to decreased phosphorylation level of AKT and mTOR proteins (Figure 5). These data suggested that RAB9A activated the AKT/mTOR signaling pathway in human liver cancer cells.

**Figure 6:**
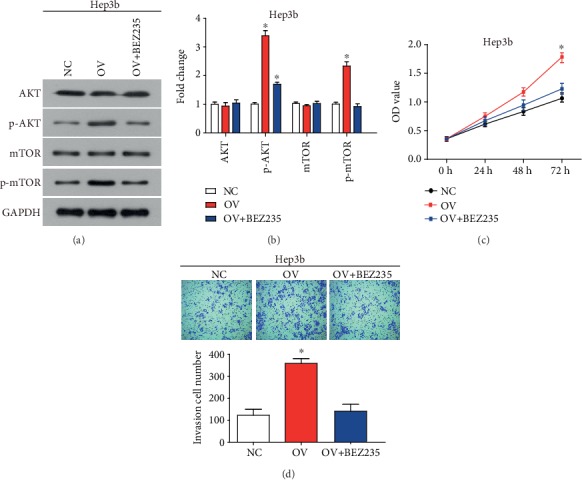
RAB9A promotes the malignant behavior of liver cancer cells by activating the AKT/mTOR signaling pathway. AKT signaling pathway components were detected (a) and quantified (b) by western blot. (c) Cell proliferation of Hep3b cells was detected by CCK8 assay. (d) The invasion of Hep3b cells was determined by transwell assay. OV: overexpression of RAB9A; OV+BEZ235: overexpression of RAB9A+treatment of double-effect inhibitor (BEZ235) which inhibits AKT and mTOR phosphorylation. ^∗^*P* < 0.05.

To reveal whether RAB9A promoted the malignant behavior of liver cancer cells by activating the AKT/mTOR signaling pathway, a double-effect inhibitor (BEZ235) which inhibits AKT and mTOR phosphorylation was used for complementation experiments. As shown in Figures 6(a) and 6(b), the treatment of BEZ235 effectively inhibited the phosphorylation of AKT and mTOR. In addition, the treatment of BEZ235 significantly hindered the effect of RAB9A overexpression on the proliferation and invasion of Hep3b cells (Figures 6(c) and 6(d)).

## 4. Discussion

In our study, we first observed that RAB9A acted as an oncogene in the progression of human liver cancer. We found that RAB9A significantly promoted the proliferation, colony formation, invasion, and migration of human liver cancer cells. In addition, overexpression of RAB9A inhibited apoptosis.

A previous study reports that RAB9A functions as an oncogene in melanoma and breast cancer cells [9, 10]. To identify the function of RAB9A in liver cancer, we used plasmid to upregulate RAB9A in Hep3b cells and used shRNA to downregulate RAB9A in HepG2 cells. CCK-8 and clone formation assay showed that RAB9A promoted the proliferation of liver cancer cells. Flow analysis showed that RAB9A inhibited apoptosis. In addition, the analysis of apoptosis-related proteins showed that the expression of the proapoptotic proteins Bax and cleaved caspase 3 was downregulated while the expression of antiapoptotic protein Bcl2 was upregulated. These results explained the mechanism by which RAB9A led to decreased apoptosis. The scratch and transwell assay suggested the inhibition of migration and invasion of HepG2 by knockdown of RAB9A. Our findings are consistent with the role of RAB9A in melanoma and breast cancer cells. Based on these results, RAB9A also plays an important oncogenic role in the progression of liver cancer.

Rabs work together with their effectors to play critical roles in regulating the vesicle-mediated transport [11]. Different Rabs can bind their specific effectors, such as the Rab4/Rab22-Rabenosyn-5 [12] and Rab6-Rab6IP1 complexes [13]. To date, several Rab9 effectors have been identified, including p40, Tip47, GCC185, and RhoBTB3, all of which are involved in MPR recycling [14–16]. In addition, a novel RAB9 interacting protein, PP2A, is identified [17]. RAB9 binds to the scaffold subunit of PP2A (PPP2R1A) and competes with the catalytic subunit of PP2A (PPP2CA) when binding. This competitive combination plays an important role in controlling the catalytic activity of PP2A. PP2A catalytic activity is impaired in some solid tumors and leukemia. Therefore, identifying proteins that interact with RAB9A in liver cancer cells is one of our future research directions and will provide new targets for the treatment of liver cancer.

Like other small GTPases, Rabs circulate between two states, the GTP-bound active state and the GDP-bound inactive state [18]. The transformation is assisted by the guanine exchange factors (GEFs) and GTPase activating proteins (GAPs) [19, 20]. However, RAB9 interacts with PPP2R1A in a GTP-independent manner [17]. Therefore, we cannot determine that the role of RAB9A in liver cancer cells is GTP-dependent or GTP-independent.

In this study, we found that RAB9A activated the AKT/mTOR signaling pathway in human liver cancer cells. Overexpression of RAB9A led to increased phosphorylation level of AKT and mTOR proteins, and RAB9A knockdown led to decreased phosphorylation level of AKT and mTOR proteins. AKT/mTOR signaling pathways are widely involved in intracellular cell proliferation, survival, protein synthesis, and other physiological activities [21]. In addition, the treatment of BEZ235 significantly hindered the effect of RAB9A overexpression on the proliferation and invasion of Hep3b cells. These data reveal that RAB9A promoted the malignant behavior of liver cancer cells by activating the AKT/mTOR signaling pathway.

## 5. Conclusions

In this study, we identify that RAB9A promotes the tumor aggressive progression of human liver cancer in vitro, which may be mediated by blocking AKT signaling pathways. Our study provides a novel perspective on the potential role of RAB9A in liver cancer therapy. In addition, little is known about the specific molecular mechanisms by which RAB9A functions in liver cancer cells.

## Data Availability

All data generated or analyzed during this study are included in this published article.

## Abbreviation

MPR: Mannose-6-phosphate receptor
TGN: *trans*-Golgi network
LIHC: Liver hepatocellular carcinoma.
